# Molecular analogue of the perovskite repeating unit and evidence for direct Mn^III^-Ce^IV^-Mn^III^ exchange coupling pathway

**DOI:** 10.1038/s41467-017-00642-0

**Published:** 2017-09-11

**Authors:** Annaliese E. Thuijs, Xiang-Guo Li, Yun-Peng Wang, Khalil A. Abboud, X.-G. Zhang, Hai-Ping Cheng, George Christou

**Affiliations:** 1 Department of Chemistry, University of Florida, Gainesville, FL 32611-7200 USA; 2 Department of Physics, University of Florida, Gainesville, FL 32611-7200 USA

## Abstract

The perovskite manganites AMnO_3_ and their doped analogues A_1–*x*_B_*x*_MnO_3_ (A and B = main group and lanthanide metals) are a fascinating family of magnetic oxides exhibiting a rich variety of properties. They are thus under intense investigation along multiple fronts, one of which is how their structural and physical properties are modified at the nanoscale. Here we show that the molecular compound [Ce_3_Mn_8_O_8_(O_2_CPh)_18_(HO_2_CPh)_2_] (Ce^III^_2_Ce^IV^Mn^III^_8_; hereafter Ce_3_Mn_8_) bears a striking structural resemblance to the repeating unit seen in the perovskite manganites. Further, magnetic studies have established that Ce_3_Mn_8_ exhibits both the combination of pairwise Mn^III^_2_ ferromagnetic and antiferromagnetic exchange interactions, and the resultant spin vector alignments that are found within the 3-D *C*-type antiferromagnetic perovskites. First-principles theoretical calculations reveal not only the expected nearest-neighbor Mn^III^_2_ exchange couplings via superexchange pathways through bridging ligands but also an unusual, direct Mn^III^–Ce^IV^–Mn^III^ metal-to-metal channel involving the Ce^IV^ *f* orbitals.

## Abstract (web summary)

Perovskite manganites exhibit intriguing but poorly understood properties, including multiferroicity. Here, the authors synthesize a Ce_3_Mn_8_ cluster that structurally resembles a perovskite repeat unit, and use this molecular analogue to elucidate mechanisms driving bulk perovskite properties.

## Introduction

Perovskite manganites continue to be a source of great interest in the scientific community owing to the fascinating and important physical properties they exhibit, such as colossal magnetoresistance and multiferroicity^1–6^. These materials have been proposed for important applications in many technological fields such as spintronics and information storage^7^. Detailed insights into the mechanisms by which these materials function has often been limited owing to their complex nature^8^. In many cases, the most direct way to understand complex systems can be to study a fragment of the larger structures, and one way to do this is to apply a molecular bottom-up approach to make 0-D species whose molecular properties can overcome many of the limitations and complexities encountered in the characterization and study of bulk 3-D materials and their nanoparticles^9^.

Molecules possess certain important advantages over nanoparticles, including particle monodispersity, crystallinity, true solubility, and a stabilizing shell of organic ligands that is also often capable of facile modification as desired. Given the success of molecular approaches to monodisperse nanoscale magnets in other areas such as single-molecule magnetism, the synthesis and study of molecules with a structural resemblance to the perovskites would provide an alternative and complementary approach to ultra-small perovskite nanoparticles. Such advantages of molecules over their bulk counterparts for study of known physical phenomena, and discovery of new ones, are well documented in the field of single-molecule magnets (SMMs)^10, 11^. These are 0-D molecular nanomagnets that possess a combination of uniaxial anisotropy and a high ground state spin, and consequently function as superparamagnets below their blocking temperatures, *T*_B_^12–16^. In a similar fashion, attainment of molecular species that combine the structural and physical properties of the perovskite manganites would provide an ideal and alternative route forward for understanding the origins of the properties of this important class of materials, including ultimately those with multiferroic behavior.

In the present work, we prepare and study a molecular compound [Ce_3_Mn_8_O_8_(O_2_CPh)_18_(HO_2_CPh)_2_] (Ce^III^_2_Ce^IV^Mn^III^_8_; hereafter Ce_3_Mn_8_) that is structurally reminiscent of the repeating unit in perovskite manganites. Magnetic characterization shows that the spin vector alignments in this molecule are the same as in the 3-D *C*-type antiferromagnetic perovskites. First-principles theoretical calculations reveal an unusual and direct Mn^III^–Ce^IV^–Mn^III^ metal-to-metal magnetic exchange channel involving the Ce^IV^ *f* orbitals, besides the conventional nearest-neighbor Mn^III^_2_ exchange couplings via superexchange pathways through bridging ligands. An excellent agreement between theory and experiment for the magnetic susceptibility curve is reached along with the establishment of an unprecedentedly rich physical picture of magnetic interaction. The work demonstrates the feasibility of a bottom-up molecular approach to gaining insights into the structural and physical properties of ultra-small nanoscale perovskite materials, as well as surface units on larger particles where structural relaxation effects and O vacancies are present.

## Results

### Synthesis and structure characteristics

**Fig. 1:**
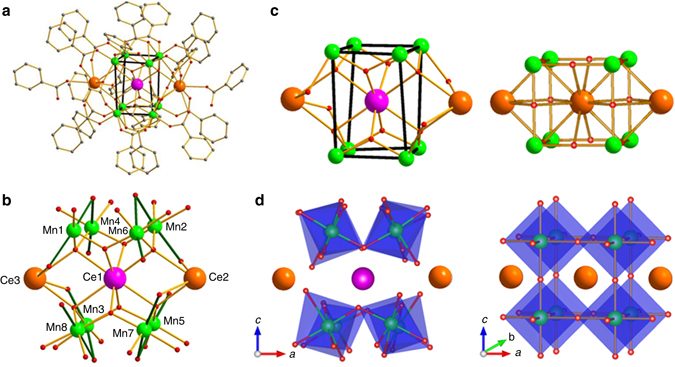
The molecular structure of Ce_3_Mn_8_ from experimental single-crystal X-ray diffractometry data. **a** The complete structure with H atoms omitted for clarity. The *thicker black lines* are guides to the eye to emphasize the distorted Mn_8_ cube. **b** The partially labeled central fragment with the Mn^III^ JT axes indicated as thicker Mn–O bonds. Color scheme: Ce^IV^ *purple*; Ce^III^ and Ln^III^ *orange*; Mn^III^ *green*; O *red*; C *gray*. **c** Comparison of the core from (**a**) of Ce_3_Mn_8_ (*left*) with a repeating unit of an ideal ABO_3_ cubic perovskite plus two A ions from adjacent repeating units (*right*). **d** As for (**c**) but showing the MnO_6_ polyhedra

3-D perovskites are prepared at high temperatures in the solid state, whereas 0-D molecules are typically prepared at or near ambient temperatures in solution. The results reported here were thus crucially dependent on the successful development of an experimental procedure for the synthesis of pure, crystalline Ce_3_Mn_8_ (see “Methods”). The structure (Fig. 1) was determined by single-crystal X-ray diffractometry (Supplementary Methods and Supplementary Tables 1–3) and consists of a {Ce^IV^Mn^III^_8_(*μ*_3_-O)_8_}^12+^ unit comprising a Mn_8_ distorted cube with a Ce^IV^ at its center held together by four *μ*_3_-O^2−^ and four *μ*_4_-O^2−^ ions, with the latter connecting to two external Ce^III^ ions attached on opposite faces of the cube. The organic ligation consists of two *μ*_4_-, four *μ*_3_-, and twelve *μ*_2_-benzoate groups, as well as two terminal benzoic acid groups on the Ce^III^ ions. All Mn^III^ atoms are six-coordinate with distorted octahedral geometries and exhibit Jahn–Teller (JT) distortion (elongation) axes. The latter are localized, meeting in pairs at the O atoms of the *μ*_4_-benzoate groups (Fig. 1b). The Ce^III^ and Ce^IV^ ions are nine-coordinate and eight-coordinate, respectively.

The core of Ce_3_Mn_8_ is overall similar to a fragment of the cubic perovskite structure, shown in Fig. 1c for comparison, where it can be seen to comprise a LnMn^III^_8_O_*x*_ (Ln = lanthanide) unit plus two additional Ln ions that in a 3-D perovskite array would be in the adjacent repeating units. It can thus be described as one complete and two partial repeating units of the perovskite structure. Also notable is that it exhibits distinct distortions from cubic symmetry and that some of these are similar to those seen in the LnMn^III^O_3_ manganites. This is particularly interesting as Ce_3_Mn_8_ is a discrete molecular complex buffered by the organic ligation from external influences, whereas many distortions within the more rigid 3-D perovskite structure are cooperative in nature. In Ce_3_Mn_8_: firstly, the cube undergoes a tetragonal distortion (stretching) along one direction due to the JT axis locations and the external Ce ions, giving short and long Mn…Mn edge separations of 3.16–3.33 Å and 4.84–4.94 Å, respectively; secondly, the MnO_6_ octahedra are tilted, allowing the bridging O^2−^ ions to move off the Mn–Mn edges and toward the center of the cube to optimize Ce–O bond lengths; and thirdly, the long Mn...Mn edges are bridged by longer PhCO_2_^−^groups rather than shorter O^2−^ bridges, so that the corresponding core is {CeMn_8_O_8_(O_2_CPh)_4_} vs {CeMn_8_O_12_} for the perovskite. The first point is reminiscent of, although of greater magnitude than the tetragonal perovskite structures, and the second point is reminiscent of the orthorhombic structure of the perovskite LnMnO_3_ manganites, where a distortion involving tilting of the MnO_6_ octahedra (by an amount dependent on the Ln ionic radius) away from the ideal cubic structure similarly arises from the combined effect of Mn^III^ JT distortions and a mismatch of the Ln–O and Mn–O bond lengths, moving the oxide ions off the Mn–Mn edges^17^. Interestingly, the above three points together suggest that Ce_3_Mn_8_ may best be considered a model for surface-repeating units of perovskite nanoparticles, where structural relaxation effects and oxide ion vacancies are present. Further comparison between Ce_3_Mn_8_ and the corresponding Ln = Ce manganite CeMnO_3_ is precluded by the absence of crystallographic information for this material^18^, which appears to be difficult to synthesize pure due to the multiple oxidation states accessible to both cerium and manganese.

### Magnetic structure analysis

**Fig. 2:**
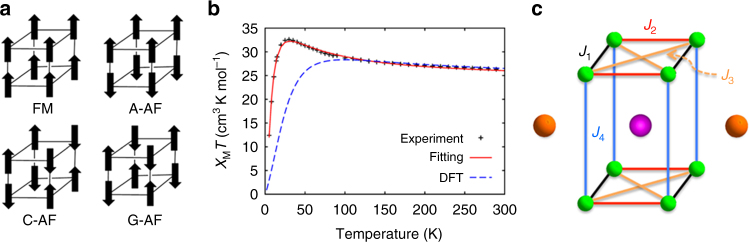
Magnetic properties of Ce_3_Mn_8_ and perovskites. **a** Four known spin-ordering configurations in a perovskite unit cell. **b** Experimental *χ*_*M*_ *T* vs *T* plot for Ce_3_Mn_8_ in a 1 kG applied dc field (+), with the small contributions from the two external Ce^III^ ions subtracted. *Solid* and *dashed lines* are theoretical results from a multi-spin Heisenberg model using either exchange coupling *J*’s and anisotropy term *D* from fits to the experimental data (*solid line*) or calculated from first-principles DFT (*dashed line*). **c** The multi-spin Heisenberg model showing the magnetic exchange coupling paths labeled as *J*_1_ to *J*_4_; *colored lines* indicate symmetry-equivalent sets. Other possible paths (unlabeled) correspond to exchange coupling strengths at least an order of magnitude smaller than the ones labeled in the figure

The location and orientation of the Mn^III^ Jahn–Teller axes in Ce_3_Mn_8_ (Fig. 1b and Supplementary Table 3) represent the local Mn *z* axes and thus the location of the singly occupied *σ*-symmetry *d*_z_^2^ orbitals. When two *d*_z_^2^ (*d*_*σ*_) orbitals meet at a bridging O atom with the acute Mn–O–Mn angles (83–88°) in Ce_3_Mn_8_, the empirical Goodenough–Kanamori (GK) rules^19–21^ predict ferromagnetic (FM) interactions for the Mn1/Mn4, Mn2/Mn6, Mn3/Mn8, and Mn5/Mn7 pairs (Fig. 1b). Exchange couplings for the other Mn^III^ pairs are expected to be dominated by overlap of *d*_*π*_ magnetic orbitals, and thus to be weakly antiferromagnetic (AF) according to the GK rules. For perovskite manganites, competition among spin, charge, and orbital degrees of freedom determine the magnetic properties, and the Mn^III^ JT axes are intimately involved^22^. Depending on the precise orbital ordering and the associated superexchange interactions in a given manganite, a particular combination of FM and AF interactions emerges^23^. The common types of spin ordering that result (ferromagnetic or FM, and three types of AF: A-AF, C-AF, and G-AF) for this structure are illustrated in the *left panel* of Fig. 2. Below we describe the experimental magnetic data for Ce_3_Mn_8_ and then we will apply first-principles electronic structure calculations to reveal the microscopic mechanics of magnetic coupling in the Ce_3_Mn_8_ molecule, thereby providing a unique angle for understanding the exchange interactions in perovskite manganites.

Experimental solid-state magnetic susceptibility (*χ*_*Μ*_) data on Ce_3_Mn_8_ were collected on a microcrystalline sample, restrained in eicosane to prevent torquing, in a 1 kG (0.1 T) field in the 5–300 K range. The obtained *χ*_*M*_ *T* vs *T* plot (*middle panel* in Fig. 2) exhibits two regimes, increasing from 26.80 cm^3 ^Kmol^−1^ at 300 K to a maximum of 32.68 cm^3 ^Kmol^−1^ at 30 K, and then steeply decreasing to 12.41 cm^3 ^Kmol^−1^ at 5 K. The value at 300 K is greater than the 25.62 cm^3 ^Kmol^−1^ calculated for eight Mn^III^ (*S* = 2, *χ*_*M*_ *T* = 3 cm^3 ^Kmol^−1^ with *g* = 2) and two Ce^III^ (*f* ^1^, *S* = ½, *L* = 3, ^2^ *F*_5/2_, *χ*_*M*_ *T* = 0.81 cm^3 ^Kmol^−1^)^24^ non-interacting ions, indicating the increasing *χ*_*M*_ *T* with decreasing *T* to be due to dominant FM pairwise Mn^III^Mn^III^ interactions within the molecule. The steep decrease below 30 K is assigned to weaker AF interactions that begin to pair-up spins at lower temperatures. *χ*_*M*_ *T* is clearly heading for a small value at ~0 K, indicating a ground state spin description for Ce_3_Mn_8_ as an AF *S* = 0 Mn_8_ cube, and two essentially non-interacting Ce^III^ ions as expected from the very weak exchange couplings exhibited by Ln^III^ ions^25^. This description is supported by the in-phase ac susceptibility (*χ′*_*M*_ *T*) vs *T* plot down to 1.8 K (Supplementary Fig. 1), which extrapolates to a small non-zero value consistent with the expected *χ*_*M*_ *T* = 1.62 cm^3 ^Kmol^−1^ for two independent Ce^III^ ions.

### First-principles energetics

To characterize the origin of the AF ground state of Ce_3_Mn_8_, the magnetic properties of Ce_3_Mn_8_ were calculated within the framework of Kohn–Sham density functional theory (DFT), using atomic coordinates for the molecule taken from the crystal structure. DFT based methods have been widely used for studying molecular magnets with Mn centers^26–31^. First-principles calculations were performed for eight different spin configurations of Ce_3_Mn_8_ molecules, corresponding to FM, A-AF, C-AF, and G-AF spin alignments (Supplementary Methods), with all three orientations of the ferromagnetic planes (A-AF) or axes (C-AF) considered and neglecting any noncolinearity (the easy axis is assumed to be along the global *z* axis). The results (Table 1) show that spin state C-AF-I has the lowest total energy. The absolute magnetization of each Mn ion is ~3.5 *μ*_B_, as expected for Mn^III^ with some induced magnetization at the O sites. Each of the two outer Ce^III^ sites has a magnetic moment of 1 *μ*_B_, which is included in the core region of the charge density. In the FM state, the central Ce^IV^ site has a small magnetic moment (0.28 *μ*_B_) and, as expected, no net spin density is found on the Ce^IV^ sites for any of the *S* = 0 AF states. The C-AF-I ground state corresponds to FM coupling in the *b* direction (i.e., the Mn1/Mn4, Mn2/Mn6, Mn3/Mn8, and Mn5/Mn7 pairs of Fig. 1b) and net AF coupling along the *a* and *c* directions, as suggested by the qualitative predictions from the structural parameters and *χ*_*M*_ *T* vs *T* data. The weak net AF coupling is supported by the low-lying A-AF-II and A-AF-III excited states, corresponding to different relative alignments of the four FM pairs. The energy difference Δ*E* between the ground state and the FM state is about 19 meV, corresponding to a switching magnetic field of *B* = Δ*E*/*gμ*_B_Δ*M* ≈ 5 T (to switch from C-AF-I to FM), where Δ*M* is the magnetic moment difference, *μ*_B_ = 0.058 me*V*/*T*, and *g* = 2. A plot of *M*/*Nμ*_B_ vs applied magnetic field (Supplementary Fig. 2) steadily increases and appears to be heading for saturation in the expected *M*/*Nμ*_B_ range of *gS* = 32 for *S* = 16, or in fact a little less as *g* < 2 slightly for Mn^III^.

**Table 1:** Total energy (*E*) and atomically resolved magnetic moments (*M* ^a^) due to valence electrons for different sites of Ce_3_Mn_8_ in different spin-ordered states

Spin order	*E* ^b,c^	abs(*M* $_{{{\bf Ce}}^{{\bf IV}}}$)^d^	*M* $_{{\bf{Ce}}^{\bf{IV}}}$	abs(*M*_Mn_)	*M*_Mn_	abs(*M*_O_)	*M*_O_	*M*_C,H_	*M*_total_
FM	19	0.28	0.28	28.57	28.57	2.97	2.97	0.17	32.00
A-AF-I	37	0.00	0.00	28.30	−0.06	2.06	0.06	0.00	0.00
A-AF-II	14	0.03	0.03	28.55	0.03	2.81	0.01	0.01	0.00
A-AF-III	10	0.03	0.00	28.22	0.04	2.17	−0.03	−0.01	0.00
C-AF-I	0	0.02	−0.02	28.20	0.04	1.99	−0.02	0.00	0.00
C-AF-II	99	0.00	0.00	27.99	−0.04	1.50	0.04	0.00	0.00
C-AF-III	19	0.02	−0.01	28.28	−0.06	1.85	0.07	0.00	0.00
G-AF	85	0.01	0.01	27.99	0.02	1.31	−0.04	0.01	0.00

^a^ *M* = *N* ^↑^−*N* ^↓^, *N* ^↑(↓)^ is the spin-up (down) charge density

^b^In meV

^c^The energy of state C-AF-I (the ground state) is set to 0

^d^abs(*M*) is the total absolute magnetization, in *μ*_B_

### Theoretical analysis of the magnetic properties

The DFT results were further analyzed using a multi-spin model, defined by the spin Hamiltonian of Eq. (1) in Supplementary Methods, to estimate the various pairwise Mn/Mn exchange coupling parameters (*J*). Four spin coupling paths (denoted by *J*_1_ through *J*_4_ in the *right panel* of Fig. 2) were found to have significant contributions to the total energy. *J*_1_ appears to involve a conventional superexchange mechanism via two monoatomically bridging oxygens. This was predicted above to be strongly FM by the GK rules. *J*_2_ appears to involve two parallel superexchange pathways: one via a single monoatomically bridging oxygen and the other through a carboxylate group (Mn–O–CR–O–Mn, R = phenyl). Both *J*_3_ and *J*_4_ appear at first glance to involve mostly superexchange through carboxylate groups; the Mn–Mn distances are 4.6 Å and 4.9 Å for the *J*_3_ and *J*_4_ pathways, respectively (compared to 3.16–3.33 Å for *J*_1_ and *J*_2_)_._ Initial calculations using the symmetry broken DFT method and incorporating *J*_1_–*J*_4_ showed that *J*_4_ was, consistent with the structure, much weaker than *J*_1_–*J*_3_ and AF. In fitting experimental data, we therefore omit *J*_4_ but include the axial anisotropy parameter (*D*) as even with current supercomputers it is not feasible to include both *D* and four *J*′s due to the excessive memory requirement. Our calculations show that the anisotropy easy axis is along the global *z* direction, perpendicular to the Mn1–Mn4–Mn2–Mn6 and Mn3–Mn5–Mn7–Mn8 planes. The calculated coupling strengths (positive for FM, negative for AF) and *D* were *J*_1_ = +1.61 meV (+13 cm^−1^), *J*_2_ = +0.72 (+5.8), *J*_3_ = −1.10 (−8.87), and *D* = −0.07 (−0.56).

The *χ*_*M*_ *T* vs *T* plot generated using the calculated DFT parameters is only in fair agreement with the experimental data (Fig. 2, *dashed line* in *middle panel*), missing the continuing rise in *χ*_*M*_ *T* below 100 K and the peak at 30 K, before the steep decrease at lower temperatures. Therefore, the *J*_1_–*J*_3_ and *D* values were refined by fitting the experimental *χ*_*M*_ *T* vs *T* data directly to the multi-spin Heisenberg model using the calculated DFT parameters as input values. An excellent fit was now obtained (Fig. 2, *solid line*) with *J*_1_ = +1.26 meV (+10.2 cm^−1^), *J*_2_ = +0.69 (+5.6), *J*_3_ = −0.74 (−6.0), and *D* = −0.05 (−0.40), again resulting in a C-AF-I ground state.

## Discussion

The above results present a consistent picture of a dominating *J*_1_ FM interaction and a C-AF ground state spin configuration. In fact, as *J*_2_ is also FM, the C-AF ground state is driven by the AF *J*_3_ coupling, the diagonal Mn_2_ interaction. It is comparable in magnitude to *J*_2_ and there is competition (spin frustration) between the *J*_1_, *J*_2_, and *J*_3_ couplings in the Mn_3_ triangles within each Mn_4_ square at the top and bottom of the molecule (Fig. 2). *J*_1_ dominates giving four FM Mn_2_ pairs, but the relative alignment of these pairs to give the C-AF-I ground state is caused by the slightly stronger *J*_3_ vs *J*_2_, i.e., |*J*_3_| > |*J*_2_|, and the AF *J*_4_. If |*J*_3_| < |*J*_2_|, however, then the ground state would have been A-AF, the A-AF-II state in Supplementary Methods. The Ce_3_Mn_8_ ground state is thus dependent on the relative values of *J*_2_ vs *J*_3_: the former is clearly mediated by the superexchange pathways through the O^2−^ and RCO_2_^−^ bridges but is opposite of the expected weakly AF character according to GK rules. Thus, we sought further insight into this unexpected result as well as the origin of the diagonal *J*_3_.

The DFT calculations allow us to analyze the microscopic electronic and magnetic processes responsible for the magnetic couplings. The sign of *J*_2_ suggests that the unoccupied Ce^IV^-4*f* orbitals residing at the upper edge of the energy gap may have an unexpectedly important role in magnetic couplings. To reveal its role, we carried out an additional DFT calculation by replacing the Ce ions in Ce_3_Mn_8_ with La ions, and made the La_3_Mn_8_ molecule negatively charged to preserve the Mn^III^ valence state. In contrast to Ce_3_Mn_8_, the La-4*f* orbitals in [La_3_Mn_8_]^−^ are ~2.5–3.5 eV above the energy gap (Supplementary Discussion), and thus are not expected to contribute to magnetic couplings. *J*_1_ to *J*_4_ in the [La_3_Mn_8_]^−^ molecule are more AF by ~0.1–0.8 meV (0.8–6.5 cm^−1^) than those in Ce_3_Mn_8_ (Supplementary Discussion). The comparison between Ce_3_Mn_8_ and La_3_Mn_8_ indicates that the contribution of Ce^IV^-4*f* orbitals in Ce_3_Mn_8_ is FM in nature, as we further explain below. The magnetic coupling among Mn^III^ ions in these molecules can be decomposed into two contributions from different physical origins: the AF superexchange coupling via bridging O^2−^ ions and/or carboxylate groups, and a FM direct exchange coupling enhanced by the Ce^IV^ ion. The strength of the superexchange coupling is determined by the effective hopping integral between Mn-*d*_z_^2^ states of two Mn ions. The integral can be calculated by downfolding the DFT Hamiltonian onto Mn-*d*_z_^2^ orbitals using the maximally localized Wannier function method^32^. The calculated effective hopping integrals in Ce_3_Mn_8_ molecule are almost the same as those in [La_3_Mn_8_]^−^ (Supplementary Discussion). From this, we conclude that the same AF superexchange couplings exist in both molecules, and rule out a possible AF superexchange coupling via Ce^IV^-4*f* orbitals. The FM direct exchange depends on the shape of Mn-3*d* orbitals. The presence of Ce^IV^-4*f* orbitals near the energy gap enhances the direct exchange coupling between neighboring Mn pairs by pulling the Mn-*d*_z_^2^ orbitals closer to each other through hybridization with the Ce^IV^-4*f* orbitals and forming an itinerant electron path between two Mn ions. Indeed, the shape of Mn-*d*_z_^2^ Wannier orbitals shows considerable difference between Ce_3_Mn_8_ and [La_3_Mn_8_]^−^ (Supplementary Discussion). The overall picture that emerges is an unexpected FM contribution to the Mn_2_ exchange couplings from a direct Mn–Ce–Mn pathway, making *J*_2_ FM and comparable in absolute magnitude to *J*_3_, leading to a C-AF ground state but with low-lying A-AF excited states. This suggests that small structural distortions (e.g., from applied pressure, changes in the identity of the ligands, etc.) could alter the *J*_2_:*J*_3_ ratio and switch the ground state to A-AF.

Overall, a synthetic method has been developed to a Ce_3_Mn_8_ cluster that shows strong structural similarity to the repeating unit of perovskite manganites. It exhibits two structural distortions common in bulk ABO_3_ perovskites, namely tilting of the BO_6_ octahedra and a tetragonal distortion driven by JT elongation of the B cations, and it also exhibits the same spin ordering as *C*-type antiferromagnetic perovskites. This demonstrates that these unusual structural and spin-ordering effects can be reproduced even at the level of a single repeating unit. We propose Ce_3_Mn_8_ may also be particularly relevant to surface units of nanoscale perovskites. First-principles-based investigations reveal the microscopic mechanism of the magnetic couplings inside the Ce_3_Mn_8_ molecule. The unoccupied Ce^IV^-4*f* orbitals have considerable contribution to the direct exchange coupling, which in turn has a pivotal role in the competition (spin frustration) between FM and AF interactions in this molecule and the resulting ground state C-AF spin configuration with low-lying A-AF excited states. These results suggest analogous effects may be important in the magnetic couplings within the extended lattices of Ce^IV^-containing perovskites or similar compounds in which 4*f* orbitals can be brought close to the Fermi energy. Attempts to complete one or both partial cubes at each end of Ce_3_Mn_8_ with additional Mn^III^ ions to yield a molecule representing two or three face-fused repeating perovskite units are in progress, as is the synthesis of Ce_3_Mn_8_ analogues with various other lanthanide or main group metal ions to expand the experimental database. New molecules of this type, analogues to the BiMnO_3_ or TbMnO_3_ systems, may even show true multiferroic bistability and further shed light on the complex mechanisms involved in these systems.

## Methods

### Experiments

The comproportionation reaction of Mn(O_2_CPh)_2_·2H_2_O, Ce(NO_3_)_3_·6H_2_O, NBu^*n*^_4_MnO_4_ and PhCO_2_H in a 4:4:1:16 molar ratio in MeNO_2_ at ~80 °C gave a dark brown solution from which was isolated [Ce_3_Mn_8_O_8_(O_2_CPh)_18_(HO_2_CPh)_2_] (Ce_3_Mn_8_) as black crystals in ~55% yield based on Mn (Supplementary Methods). Single-crystal X-ray diffraction studies at 100 K were performed on a Bruker DUO diffractometer using MoK_*α*_ (*λ* = 0.71073 Å) or CuK_*α*_ (*λ* = 1.54178 Å) radiation (from an ImuS power source), and an APEXII CCD area detector. The metal oxidation states and the oxygen protonation levels were confirmed by charge considerations and bond valence sum calculations (Supplementary Tables 1, 2)^33, 34^.

### Computations

Electronic and magnetic properties of the Ce_3_Mn_8_ molecule were calculated within the framework of Kohn–Sham DFT^35^ using the spin-polarized Perder–Burke–Ernzehof^36^ exchange correlation functional and project-augmented wave^37, 38^ pseudopotentials in conjuction with the plan-wave basis as implemented in the Vienna Ab-initio Simulation Package^39, 40^. The plane-wave cutoff energy was 500 eV, and the energy threshold for self-consistency was 10^−5^ eV. Owing to the strong localization of the Ce *f* electron, the GGA + *U* method was applied with *U* = 2 eV^41^ for the Ce *f* orbitals. Spin–orbit interactions were also included.

### Data availability

The crystallographic information file (CIF) for [CeMn_8_O_8_(O_2_CPh)_18_(HO_2_CPh)_2_]·*x*(solvent) (Ce_3_Mn_8_) has been deposited at the Cambridge Crystallographic Data Centre with deposition code CCDC 1533475.

## Electronic supplementary material

Supplementary Information
