# Supplementary material for: Molecular analogue of the perovskite repeating unit and evidence for direct MnIII-CeIV-MnIII exchange coupling pathway
Source: Nat Commun. 2017 Sep 11;8:500. doi: 10.1038/s41467-017-00642-0 (PMC5593820; doi:10.1038/s41467-017-00642-0)
Supplement: Supplementary file 1 — Supplementary Information [file 41467_2017_642_MOESM1_ESM.pdf]

File Name: Supplementary Information
Description: Supplementary Methods, Supplementary Tables, Supplementary Figures, Supplementary Discussion, Supplementary References.

Supplementary Methods

Synthesis of [$Ce^{III}_2Ce^{IV}Mn^{III}_8O_8(O_2CPh)_{18}(HO_2CPh)_2$] ($Ce_3Mn_8$)

Solid $PhCO_2H$ (1.00 g, 8.20 mmol) was dissolved in $MeNO_2$ (20 mL) at 80 ºC with stirring, and the resulting colourless solution was treated with solid $Mn(O_2CPh)_2 \cdot 2H_2O$ (0.700 g, 2.05 mmol) which resulted in a slightly pink solution. Upon addition of $Ce(NO_3)_3 \cdot 6H_2O$ (0.910 g, 2.05 mmol), the solution colour changed rapidly to a deep red. The solution was stirred at 80°C for 5 min during which time solid $NBu^n_4MnO_4$ (0.19 g, 0.53 mmol) was added in small portions. The resulting dark brown solution was stirred for a further hour and filtered hot. The filtrate was left to cool and filtered again once the solution reached room temperature to remove some white benzoic acid crystals that formed. The final solution was left undisturbed for four days during which time X-ray quality dark-brown, block-like crystals of [$Ce_3Mn_8O_8(O_2CPh)_{18}(HO_2CPh)_2$]·*x*(solvent) slowly grew. These were collected by filtration, washed with cold $MeNO_2$, and dried under vacuum. Yield = 55 % based on Mn. The vacuum-dried solid analyzed as solvent-free. Anal. Calcd (Found) for $C_{140}H_{102}Ce_3Mn_8O_{48}$: C, 49.28 (49.08); H, 3.01 (3.06); N, 0.00 (0.02). Selected IR data (KBr, cm^{-1}): 3423 (w), 3062 (w), 1691 (w), 1601 (m), 1568 (m), 1545 (s), 1493 (m), 1412 (vs), 1307 (m), 1177 (m), 1156 (m), 1068 (m), 1025 (m), 1002 (w), 937 (w), 842 (w), 815 (w), 716 (s), 684 (m), 608 (m), 581 (m), 529 (m), 463 (w), 426 (w).

X-ray Crystal Structure

Crystal structure data for Ce_3Mn_8: $C_{140}H_{102}Ce_3Mn_8O_{48}$ (excluding solvent of crystallization), M_r = 3412.24, triclinic, space group $P\bar{1}$, a = 15.3010(6) Å, b = 17.1377(7) Å, c =28.1603(11) Å, α = 91.9495(7)°, β = 102.8680(7)°, γ = 97.9738(7), V = 7113.1(5) Å^3, Z = 2, ϱ_{calc} = 1.646 g/cm^3, T = 100(2) K, 32685 reflections (of which 27321 are observed with $I > 2\sigma(I)$) were used to refine 1649 parameters and the resulting R_1, wR_2 and S (goodness of fit) were 5.08%, 12.17% and 1.071, respectively. X-Ray intensity data were collected on a Bruker DUO diffractometer using Mo Kα radiation (λ = 0.71073 Å), from an ImuS power source, and an APEXII CCD area detector. The asymmetric unit consists of the whole Ce_3Mn_8 molecule and some disordered solvent molecules of crystallization. Several benzoate ligands on the Ce_3Mn_8

molecule were disordered: five had their phenyl rings fully disordered between two positions, and two ligands had partially disordered phenyl rings.

DFT Calculation: Method and Magnetic Structure

Electronic and magnetic properties of the Ce_3Mn_8 molecule were calculated within the framework of Kohn-Sham density functional theory (DFT) [1] using the spin-polarized Perder-Burke-Ernzehof (PBE) exchange correlation functional [2] and project-augmented wave (PAW) [3] [4] pseudopotentials in conjunction with the plan-wave basis as implemented in the Vienna Ab-initio Simulation Package (VASP) [5] [6]. The plane-wave cutoff energy is 500 eV, the energy threshold for self-consistency is 10^{-5} eV. Due to the strong localization of the Ce f electron, we apply GGA+U method with $U = 2$ eV [7] for the Ce f orbitals. Spin-orbit interaction (SOI) are also included in our calculations.

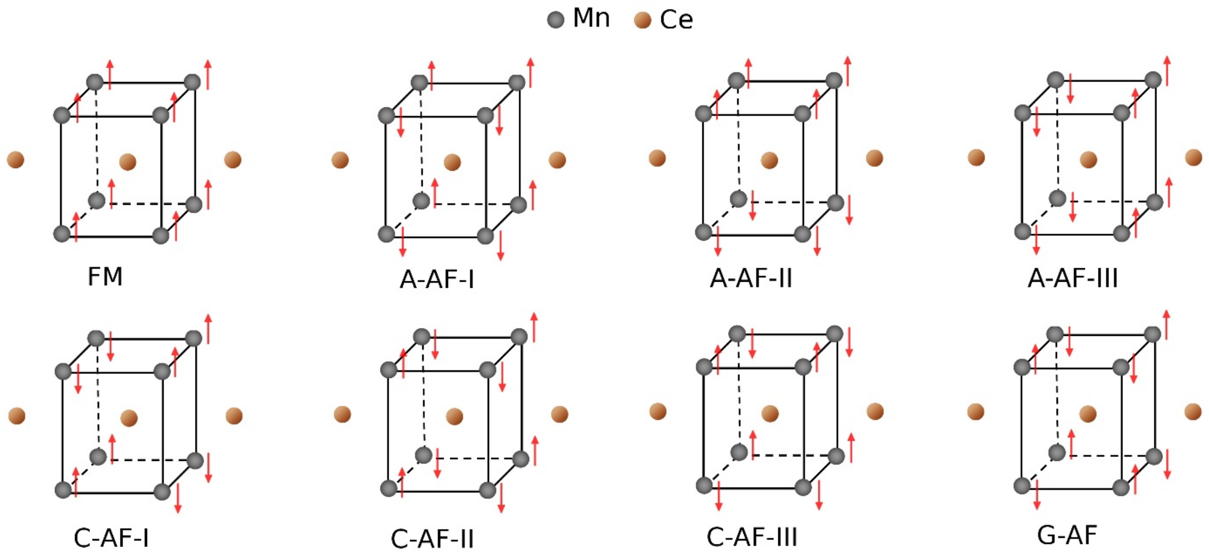

Spin vector alignment in total energy calculations of the eight Mn^{III} spin configurations. Red arrows indicate the spin direction at each Mn ion.

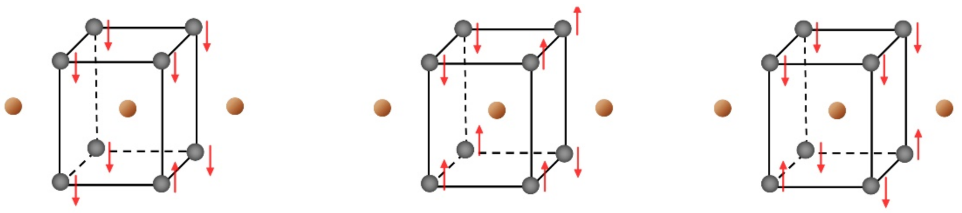

Three additional spin configurations for Ce_3Mn_8 molecule for total energy calculations to fit J values. Red arrows indicate the spin direction at each Mn ion.

A multi-spin Heisenberg model is used as a tool to interpret the experimental susceptibility data and the first-principles total energies. The symmetry-broken approach [8] [9] [10] [11] [12] builds up the relationship between first-principles total energies and the exchange coupling constants in the Heisenberg model. The four 3*d* electrons on each Mn ion have spins parallel to each other according to the Hund's rule, so the total spin *S*=2. The multi-spin Hamiltonian is,

$$\hat{H} = - \sum_{i<j} J_{ij} \vec{S}_{\mathrm{i}} \cdot \vec{S}_{\mathrm{j}} \,, \qquad (1)$$

where the summation is over all distinct spin pairs, $\vec{S}_{\mathrm{i}}$ is the spin vector on site i, and J_{ij}, is the exchange-coupling between the moments on sites *i* and *j*. With four coupling *J* parameters, J_1, J_2, J_3, J_4 in the right panel of Figure 2, both the magnetic susceptibility curve and the first-principles total energies for different spin configurations can be reasonably reproduced. Fitting to experimental (first-principles) data yields J_1=1.50 (1.61), J_2=1.24 (0.72), J_3=-1.03 (-1.10), J_4=-0.14 (-0.30), in meV.

Supplementary Table 1

Bond Valence Sums[a] for the Mn and Ce atoms in Ce_3Mn_8.

Atom[a]	Mn^{II}	Mn^{III}	Mn^{IV}	Atom[b]	Ce^{III}	Ce^{IV}
Mn1	3.18	**2.91**	3.06	Ce1	4.41	**3.97**
Mn2	3.19	**2.92**	3.07	Ce2	**3.12**	2.80
Mn3	3.17	**2.90**	3.05	Ce3	**3.18**	2.87
Mn4	3.17	**2.90**	3.04			
Mn5	3.19	**2.92**	3.07			
Mn6	3.22	**2.95**	3.09			
Mn7	3.20	**2.93**	3.07			
Mn8	3.28	**3.00**	3.15			

[a] The oxidation state can be taken as the nearest whole number to the bold value, which is the one closest to the charge for which it was calculated.

Supplementary Table 2

Bond Valence Sums and Assignments for Selected O Atoms in Ce_3Mn_8.

Atom[a]	BVS	Assignment
O1	2.11	O^{2-}
O2	2.01	O^{2-}
O3	2.22	O^{2-}
O4	2.14	O^{2-}
O5	2.15	O^{2-}
O6	1.97	O^{2-}
O7	2.03	O^{2-}
O8	1.98	O^{2-}
O16	1.14	OH^-
O22	1.19	OH^-

[a] An O BVS in the ~1.8-2.0, ~1.0-1.2 and ~0.2-0.4 ranges is indicative of non-, single- and double-protonation, respectively.

Supplementary Table 3

Selected interatomic distances (Å)[a] and angles (deg)

Ce1-O5	2.231(3)	Mn2-O3	1.858(3)	Mn8-O46	1.929(4)
Ce1-O4	2.237(3)	Mn2-O2	1.912(3)	Mn8-O30	1.942(4)
Ce1-O3	2.257(3)	Mn2-O14	1.940(3)	**Mn8-O24**	**2.148(4)**
Ce1-O1	2.270(3)	Mn2-O39	1.948(3)	**Mn8-O26**	**2.403(4)**
Ce1-O8	2.484(3)	**Mn2-O28**	**2.234(4)**	Mn6-O1-Mn1	125.70(19)
Ce1-O6	2.486(3)	**Mn2-O31**	**2.303(3)**	Mn6-O1-Ce1	108.26(13)
Ce1-O2	2.497(3)	Mn2-Mn6	3.1508(10)	Mn1-O1-Ce1	107.09(14)
Ce1-O7	2.527(3)	Mn3-O4	1.869(3)	Mn6-O2-Mn2	111.35(16)
Ce1-Mn7	3.3247(8)	Mn3-O10	1.933(3)	Mn6-O2-Ce1	98.54(12)
Ce1-Mn5	3.3330(8)	Mn3-O7	1.938(3)	Mn2-O2-Ce1	97.73(12)
Ce1-Mn1	3.3370(8)	Mn3-O34	1.964(4)	Mn6-O2-Ce2	125.29(14)
Ce1-Mn2	3.3427(8)	**Mn3-O47**	**2.189(4)**	Mn2-O2-Ce2	110.82(13)
Ce2-O19	2.460(4)	**Mn3-O26**	**2.271(4)**	Ce1-O2-Ce2	108.58(12)
Ce2-O43	2.465(3)	Mn3-Mn8	3.1627(12)	Mn2-O3-Mn4	125.45(19)
Ce2-O6	2.479(3)	Mn4-O3	1.879(3)	Mn2-O3-Ce1	108.26(14)
Ce2-O38	2.493(4)	Mn4-O8	1.911(3)	Mn4-O3-Ce1	107.77(14)
Ce2-O2	2.501(3)	Mn4-O17	1.952(3)	Mn3-O4-Mn5	125.53(19)
Ce2-O21	2.587(3)	Mn4-O13	1.958(4)	Mn3-O4-Ce1	109.81(14)
Ce2-O31	2.597(3)	**Mn4-O48**	**2.160(4)**	Mn5-O4-Ce1	108.07(15)
Ce2-O40	2.662(3)	**Mn4-O27**	**2.324(4)**	Mn7-O5-Mn8	126.3(2)
Ce2-O41	2.695(3)	Mn5-O4	1.873(3)	Mn7-O5-Ce1	108.37(14)
Ce2-Mn2	3.6483(8)	Mn5-O6	1.910(3)	Mn8-O5-Ce1	109.27(14)
Ce2-Mn7	3.6543(8)	Mn5-O20	1.937(3)	Mn5-O6-Mn7	112.09(16)
Ce3-O18	2.426(3)	Mn5-O33	1.953(4)	Mn5-O6-Ce2	125.05(14)
Ce3-O7	2.458(3)	**Mn5-O32**	**2.125(4)**	Mn7-O6-Ce2	110.88(13)
Ce3-O9	2.461(4)	**Mn5-O25**	**2.443(4)**	Mn5-O6-Ce1	97.75(12)
Ce3-O8	2.480(3)	Mn5-Mn7	3.1966(11)	Mn7-O6-Ce1	96.53(12)
Ce3-O45	2.500(4)	Mn6-O1	1.861(3)	Ce2-O6-Ce1	109.66(13)
Ce3-O23	2.613(4)	Mn6-O2	1.903(3)	Mn8-O7-Mn3	112.09(17)
Ce3-O15	2.629(3)	Mn6-O44	1.931(3)	Mn8-O7-Ce3	124.50(15)
Ce3-O11	2.646(4)	Mn6-O35	1.950(3)	Mn3-O7-Ce3	111.47(14)
Ce3-O47	2.657(3)	**Mn6-O42**	**2.117(4)**	Mn8-O7-Ce1	98.28(13)
Ce3-Mn1	3.6346(8)	**Mn6-O28**	**2.516(4)**	Mn3-O7-Ce1	96.99(12)
Ce3-Mn3	3.6451(8)	Mn7-O5	1.860(3)	Ce3-O7-Ce1	108.77(13)
Mn1-O1	1.868(3)	Mn7-O37	1.937(3)	Mn4-O8-Mn1	110.49(17)
Mn1-O8	1.936(3)	Mn7-O6	1.943(3)	Mn4-O8-Ce3	126.37(15)
Mn1-O12	1.946(4)	Mn7-O29	1.965(3)	Mn1-O8-Ce3	110.18(13)
Mn1-O36	1.952(4)	**Mn7-O25**	**2.198(4)**	Mn4-O8-Ce1	98.42(12)
**Mn1-O27**	**2.223(4)**	**Mn7-O41**	**2.228(4)**	Mn1-O8-Ce1	97.28(12)
**Mn1-O23**	**2.223(4)**	Mn8-O7	1.875(3)	Ce3-O8-Ce1	109.45(13)
Mn1-Mn4	3.1604(11)	Mn8-O5	1.876(3)		

[a] Mn^{III} Jahn-Teller elongated bonds are shown in bold.

Supplementary Figure 1

In-phase ac susceptibility ($\chi'_{M}T$) vs T for Ce_3Mn_8 in the 1.8-15 K temperature range and in a 3.5 G ac field at a 1000 Hz ac frequency and zero dc field.

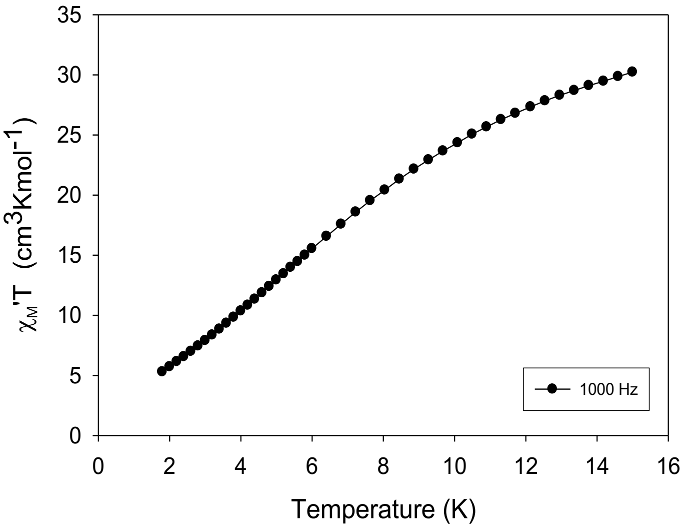

Supplementary Figure 2

Plot of reduced magnetization $MN\mu_B$ vs applied magnetic field for Ce_3Mn_8, where M is magnetization, N is Avogadro's number, and μ_B is the Bohr magneton. Reduced magnetization is the magnetization per molecule, in units of μ_B.

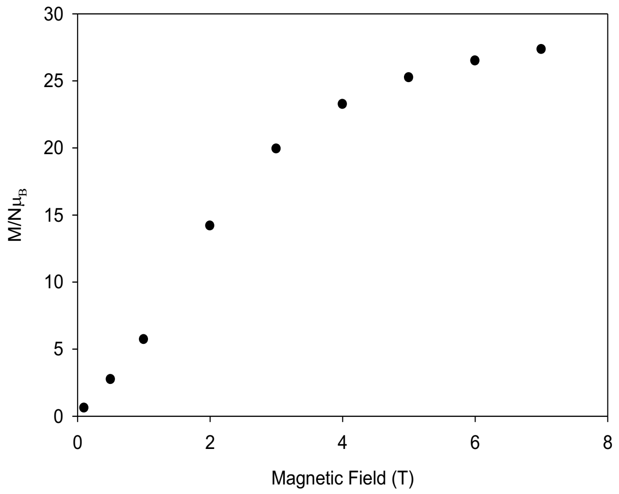

Supplementary Discussion

We use La_3Mn_8 as a model system to be compared with the Ce_3Mn_8 system. We replace the Ce ions with La ions, keep the same structure, and add one electron to the system to keep the total electrons and valence states of Mn ions the same as the Ce_3Mn_8 molecule. In the following text, La_3Mn_8 will be referred to as the charged system.

Exchange-coupling parameters and spin anisotropy

To demonstrate the validity of the cutoff of the exchange coupling parameters, we consider additional coupling parameters J_5, as the coupling between the diagonal Mn pairs of the left and right faces in the right panel (i.e., the Mn1/Mn3, Mn4/Mn8, Mn2/Mn7 and Mn5/Mn6 pairs of Figure 1), and J_6, as the coupling between the diagonal Mn pairs of the front and back faces (i.e., the Mn4/Mn5, Mn2/Mn3, Mn1/Mn7 and Mn6/Mn8 pairs of Figure 1). The fitted values from DFT total energies (the Table below) show that J_5 and J_6 are about one order of magnitude smaller than J_1-J_4. In addition, First-principles calculations show that the anisotropy exists in the molecule. The calculated anisotropy parameter D is included in the Table. The following Table also shows the fitted parameters (J_1-J_3 and D) from experimental susceptibility curve.

Fitted exchange-coupling parameters (in meV) from DFT total energies and the experimental susceptibility curve for both Ce_3Mn_8 and La_3Mn_8 (only DFT results) using the multi-spin Heisenberg model.

Exchange-coupling parameters	Ce_3Mn_8 (DFT)	La_3Mn_8 (DFT)	Ce_3Mn_8 (Fit to EXP)
J_1	1.61	0.82	1.26
J_2	0.72	0.01	0.69
J_3	-1.10	-1.42	-0.74
J_4	-0.30	-0.45	-
J_5	0.07	-0.09	-
J_6	0.04	-0.06	-
D	-0.07	-0.06	-0.05

Projected density of states (PDOS)

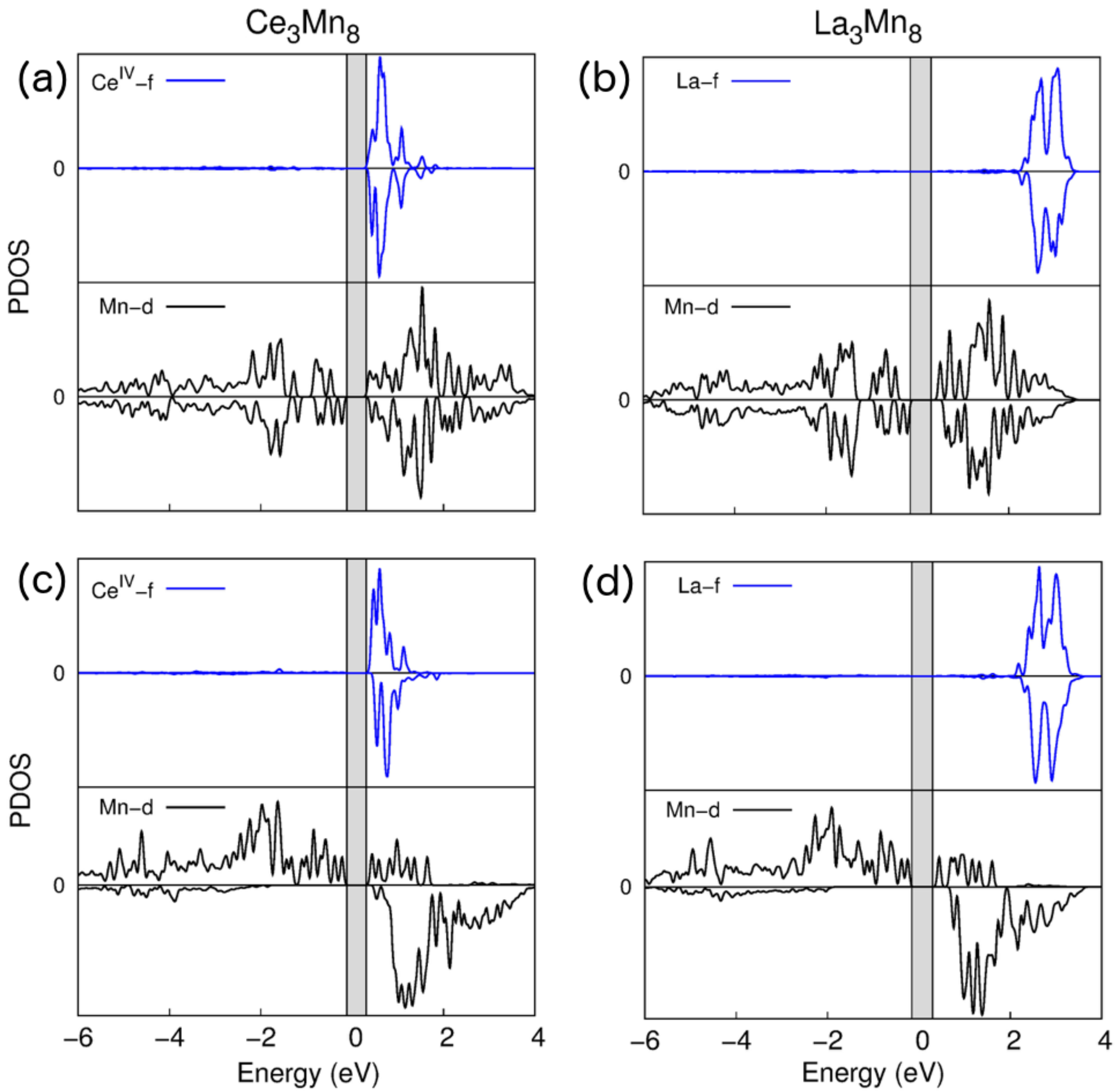

Projected density of states to Mn-*d* and Ce^{IV}-*f* or La-*f* orbitals for both (a,c) Ce_3Mn_8 and (b,d) La_3Mn_8 molecules in (a,b) C-AF-I and (c,d) FM states. The HOMO-LUMO gap is denoted by the shaded area. The main difference between these two molecules is the location of the *f* orbital. In Ce_3Mn_8, energy levels of the middle Ce^{IV} ion *f* orbitals are just above the LUMO, while in La_3Mn_8, the *f* orbital is about 2 to 3 eV above LUMO.

Wannier orbitals and downfolding

Downfolding procedure refers to the construction of an effective Hamiltonian that reproduces the original energy levels (or energy bands in solids) in a given energy range, typically around the HOMO-LUMO gap (or the Fermi energy in a solid). Matrix elements of the effective Hamiltonian are calculated as $< W_n|\hat{H}|W_m >$, where $\hat{H}$ is the Hamiltonian and $|W >$ are spatially localized orbitals, or the so-called Wannier functions generated from unitary transformation of Block waves [13].

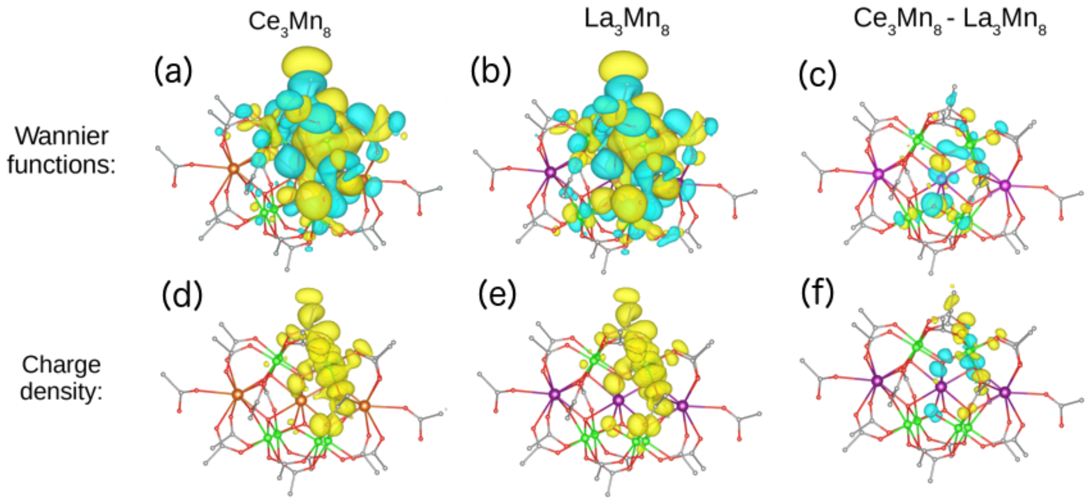

Contour plots of the Wannier orbital onto Mn-d_{z^2} subspace for Ce_3Mn_8 (a) and La_3Mn_8 (b) with isovalues cyan, and difference between them (c); isovalues: cyan ($-1.5/\sqrt{V}$), yellow ($1.5/\sqrt{V}$). V is the unit cell volume. H atoms are omitted for clarity. (d-f): the corresponding charge density and charge density difference.

We start the downfolding procedure with a DFT calculation of the ferromagnetic configuration in which all magnetic moments on Mn ion are pointing in the "up" direction. To determine the downfolding atomic basis, we need to analyze the corresponding PDOS for spin-up channel of the FM state. We are interested in two energy windows that are close to the HOMO-LUMO gap. They are the LUMO region, which is an energy window from the LUMO to ~2 eV energy above and contains mostly the *f*-orbitals from the Ce^{IV} ion (for Ce_3Mn_8 only) and the high-energy *d*-

orbitals, $d_{x^2-y^2}$ from the Mn ions, and the HOMO region, which is an energy window from the HOMO to ~1eV below and contains mostly the Mn-d_{z^2} orbitals. To reproduce the energy levels in the HOMO region, we include the 8 Mn-d_{z^2} atomic orbitals in the downfolding procedure for both Ce_3Mn_8 and La_3Mn_8. For Ce_3Mn_8, after unitary transformation, the Wannier orbitals are made from the atomic Mn-d_{z^2} orbital (HOMO region), Ce^{IV}-*f* and Mn-$d_{x^2-y^2}$ orbitals (LUMO region). For La_3Mn_8, the Wannier orbitals are made from the atomic Mn-d_{z^2} (HOMO region), and Mn-$d_{x^2-y^2}$ orbitals (LUMO region). The difference between the Wannier orbitals for Ce_3Mn_8 and La_3Mn_8 reflects the role of the Ce^{IV}-*f* orbitals in Ce_3Mn_8.

The magnetic coupling constant between each pair of Mn ions contains an AFM super-exchange contribution, and a FM direct exchange contribution. [14] The AFM contribution depends mainly on the hopping integral in the downfolded Hamiltonian. The following Table lists the hopping integral between the Mn sites for each *J* path in both Ce_3Mn_8 and La_3Mn_8 molecules. We can see that the hopping integrals are very similar between these two systems, indicating that the AFM couplings between the Mn sites are not very different in the two systems. From this we conclude that the difference in *J*'s between Ce_3Mn_8 and La_3Mn_8 is due to the difference in the FM direct exchange contribution. The latter depends sensitively on the shapes of the Wannier functions of the Mn-d_{z^2} orbitals. One of these Wannier functions and its corresponding charge density in Ce_3Mn_8 and La_3Mn_8, as well as their differences, are plotted in the above Figure. The significant difference between the Wannier function of the same orbital on both molecules points to the

significant impact of the *f*-orbitals from the Ce^{IV} ion. The charge density on the carboxylate group in the Ce_3Mn_8 system is increased, which enhances the direct exchange coupling between the d_{z^2} orbitals of neighboring Mn ions.

Hopping t_{ij} between Mn-d_{z^2} Wannier orbitals. The index of Mn sites are the same as Figure 1.

J paths	Mn sites	Ce_3Mn_8 t_{ij} (meV)	La_3Mn_8 t_{ij} (meV)
J_1 path	2-6	-15	-29
	1-4	-17	-25
	5-7	9	7
	8-3	31	20
J_2 path	2-4	47	45
	1-6	-162	-160
	5-3	-109	-115
	8-7	3	1.6
J_3 path	2-1	70	55
	4-6	107	99.8
	5-8	112	109

	3-7	96	92.5
J_4 path	2-5	7	1.8
	1-8	5	0
	4-3	57	51.6
	6-7	65	63.5
J_5 path	2-7	-33	-35.5
	6-5	11	-3.3
	1-3	-33	-39.6
	4-8	16	10
J_6 path	2-3	-2	5
	4-5	-2	-3
	1-7	4	13
	6-8	-5	-8.6

Supplementary References

[1] W. Kohn and L. J. Sham, Self-consistent equations including exchange and correlation effects, *Phys. Rev.,* 140, A1133-A1138 (1965).

[2] J. P. Perdew, K. Burke and M. Ernzerhof, Generalized gradient approximation made simple, *Phys. Rev. Lett.,* 77, 3865-3868 (1996).

[3] P. E. Blochl, Projector augmented-wave method, *Phys. Rev. B,* 50, 17953-17979 (1994).

[4] G. Kresse and J. Joubert, From ultrasoft pseudopotentials to the projector augmented wave method, *Phys. Rev. B,* 59, 1758-1775 (1999).

[5] G. Kresse and J. Furthmuller, Efficiency of an-initio total energy calculations for metals and semiconductors using a plane-wave basis set, *Comput. Mater. Sci.,* 6, 15-50 (1996).

[6] G. Kresse and J. Furthmuller, Efficient iterative schemes for ab initio total-energy calculations using a plane-wave basis set, *Phys. Rev. B,* 54, 11169-11186 (1996).

[7] C. Loschen, J. Carrasco, K. M. Neyman and F. Illas, First-principles LDA+U and GGA+U study of cerium oxides: dependence on the effective U parameter, *Phys. Rev. B,* 75, 035115 (2007).

[8] L. Noodleman, Valence bond description of antiferromagnetic coupling in transition metal dimers, *J. Chem. Phys.,* 74, 5737-5743 (1981).

[9] L. Noodleman and E. R. Davidson, Ligand spin polarization and antiferromagnetic coupling in transition metal dimers, *Chemical Physics,* 109, 131-143 (1986).

[10] L. Noodleman, C. Y. Peng, D. A. Case and J.-M. Mouesca, Orbital interactions, e;ectron delocalization and spin coupling in iron-sulfur clusters, *Coordination Chemistry Reviews,* 144, 199-244 (1995).

[11] K. Yamaguchi, T. Fueno, N. Ueyama, A. Nakamura and M. Ozaki, Antiferromagnetic spin couplings between iron ions in iron-sulfur clusters. A localized picture by the spin vector model, *Chemical Physics Letters,* 164, 210-216 (1989).

[12] K. Yamaguchi, T. Tsunekawa, Y. Toyoda and T. Fueno, Ab initio molecular orbital calculations of effective exchange integrals between transition metal ions, *Chemical Physics Letters,* 143, 371-376 (1988).

[13] M. Marzari, A. A. Mostofi, J. R. Yates, I. Souza and D. Vanderbilt, Maximally localized Wannier functions: theory and applications, *Rev. Mod. Phys.,* 84, 1419-1475 (2012).

[14] Z. Liu, J.-W. Mei and F. Liu, First-principles study of the organometallic S=1/2 kagome compound Cu(1,3-bdc), *Phys. Rev. B,* 92, 165101 (2015).
